# Rietveld refinement of the crystal structures of Rb_2_ *X*Si_5_O_12_ (*X* = Ni, Mn)

**DOI:** 10.1107/S2056989016001390

**Published:** 2016-01-27

**Authors:** Anthony M. T. Bell, C. Michael B. Henderson

**Affiliations:** a Materials and Engineering Research Institute (MERI), Sheffield Hallam University, Sheffield S1 1WB, England; b School of Earth, Atmospheric and Environmental Sciences, University of Manchester, Manchester M13 9PL, England; c ASTeC, Sci-Tech Daresbury Laboratory, Science and Technology Facilities Council, Warrington WA4 4AD, England

**Keywords:** powder diffraction, leucite minerals, silicate framework minerals, cation-ordered structure

## Abstract

The synthetic leucite silicate framework mineral analogues Rb_2_ *X*Si_5_O_12_ {*X* = Ni [dirubidium nickel(II) penta­silicate] and Mn [dirubidium manganese(II) penta­silicate]} have been prepared by high-temperature solid-state synthesis. The results of Rietveld refinements, using X-ray powder diffraction data collected using Cu *K*α X-rays, show that the title compounds crystallize in the space group *Pbca* and adopt the cation-ordered structure of Cs_2_CdSi_5_O_12_ and other leucites. The structures consist of tetra­hedral SiO_4_ and *X*O_4_ units sharing corners to form a partially substituted silicate framework. Extraframework Rb^+^ cations sit in channels in the framework. All atoms occupy the 8*c* general position for this space group. In these refined structures, silicon and *X* atoms are ordered onto separate tetra­hedrally coordinated sites (T-sites). However, the Ni displacement parameter and the Ni—O bond lengths suggest that for the *X* = Ni sample, there may actually be some T-site cation disorder.

## Abstract 2

Rietveld refinements show that the crystal structures of synthetic leucite silicate framework mineral analogues Rb_2_ *X*Si_5_O_12_ (*X* = Ni, Mn) are isostructural with the *Pbca* cation-ordered structure of Cs_2_CdSi_5_O_12_.

## Chemical context   

Synthetic analogues of the silicate framework minerals leucite KAlSi_2_O_6_ (Mazzi *et al.*, 1976 ▸) and pollucite CsAlSi_2_O_6_ (Dimitrijevic *et al.*, 1991 ▸) can be prepared with the general formulae *AB*Si_2_O_6_ and *A*_2_ *C*Si_5_O_12_. *A* is an alkali metal cation (K, Rb, Cs), *B* is a trivalent cation (Al, B, Fe^3+^) and *C* is a divalent cation (Be, Mg, Mn, Fe^2+^, Co, Ni, Cu, Zn, Cd). The title compounds are leucite analogues with *A* = Rb and *C* = Ni and Mn, these structures are in the space group *Pbca* and are isostructural with Cs_2_CdSi_5_O_12_ (Bell *et al.*, 1994*b* ▸).

These leucite structures all have the same topology, a silicate framework structure with *B* or *C* cations partially substituting on the tetra­hedrally coordinated silicon sites (T-sites). *A* cations sit in the extraframework channels, these extraframework cations can be removed by ion exchange which makes them of technological inter­est as a possible storage medium for radioactive Cs from nuclear waste (Gatta *et al.*, 2008 ▸).

## Structural commentary   

For the *X* = Ni refinement, the Ni site isotropic temperature factor was larger than expected [*B*_iso_ = 7.5 (9) Å^2^]. Also the mean Ni—O bond length for the NiO_4_ tetra­hedron is 1.90 (2) Å, shorter than that seen in tetra­hedrally coordinated NiO_4_ units. NiCr_2_O_4_ has the cubic spinel structure with Ni in tetra­hedral coordination. A single-crystal structure refinement (Crottaz *et al.*, 1997 ▸) gives the Ni—O distance as 1.967 (3) Å. An EXAFS/XANES study (Farges *et al.*, 2001 ▸) gives the Ni—O distance as 1.96 (1) Å. The mean Si—O bond length for the SiO_4_ tetra­hedra in the title structure is 1.643 (18) Å, which is slightly larger than the range of Si—O distances for silicates [1.59–1.63 Å; *Inter­national Tables for X-ray Crystallography* (1975, Vol. III, Table 4.1.1)]. These differences in the Ni—O and Si—O distances suggest some possible T-site cation disorder with some Si on the Ni site. A future higher quality neutron/synchrotron X-ray powder diffraction study may show if there really is Ni/Si T-site cation disorder.

**Figure 1:**
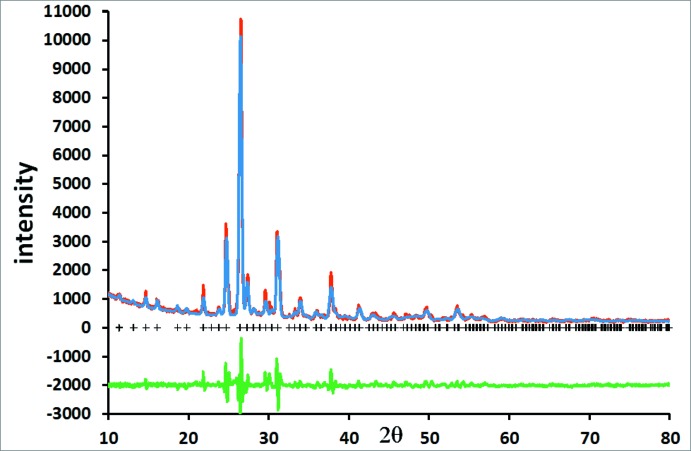
Rietveld difference plot for the single-phase refinement of Rb_2_NiSi_5_O_12_. The red, blue and green lines show, respectively, the observed, calculated and difference plots. Calculated Bragg reflection positions are indicated by crosses.

**Figure 2:**
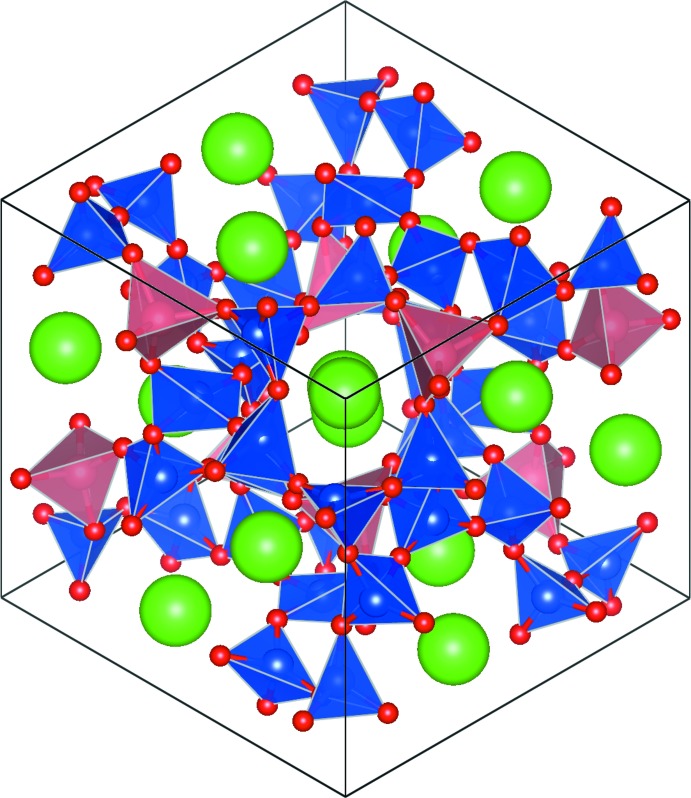
The crystal structure of Rb_2_NiSi_5_O_12_. Green spheres show Rb cations, blue polyhedra show SiO_4_ units, pink polyhedra show NiO_4_ units and red spheres represent O atoms.

Fig. 1 ▸ shows the Rietveld difference plot for Rb_2_NiSi_5_O_12_. The crystal structure of Rb_2_NiSi_5_O_12_ is displayed in Fig. 2 ▸ and consists of a framework of corner-sharing tetra­hedral SiO_4_ and NiO_4_ units, and Rb^+^ cations sitting in the extraframework channels.

For the *X* = Mn refinement, unlike in the *X* = Ni sample, the mean Mn—O distance is 2.02 (1) Å. This is in agreement with the mean Mn—O distance for Cs_2_MnSi_5_O_12_ (1.98 (3) Å; Bell & Henderson, 1996 ▸). This would suggest that this structure has complete T-site cation ordering. However, the isotropic temperature factors for the Mn site and the O sites could not be refined to chemically sensible positive values, so these were both fixed at *B*_iso_ = 0.1 Å^2^. This refinement was done assuming that Mn was present as Mn^2+^ and that all sites were fully occupied. If some or all of the Mn atoms were present with a higher oxidation state, then this could account for the problem with refining these temperature factors. However, a higher quality neutron/synchrotron X-ray powder diffraction study may also be needed for a more precise determination of the state of Mn in this structure.

**Figure 3:**
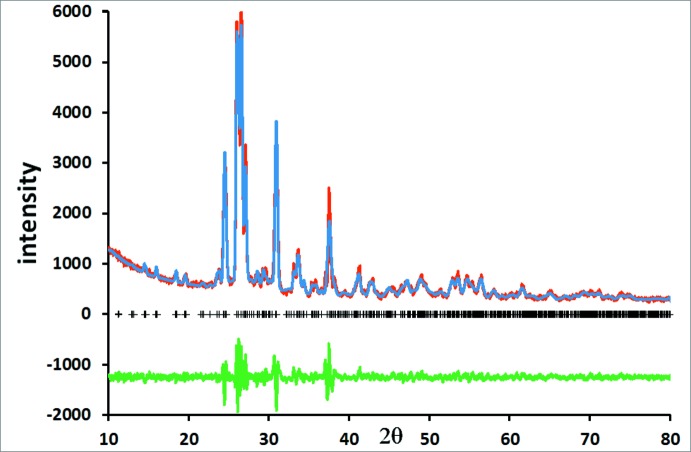
Rietveld difference plot for the single-phase refinement of Rb_2_MnSi_5_O_12_. The red, blue and green lines show, respectively, the observed, calculated and difference plots. Calculated Bragg reflection positions are indicated by crosses.

**Figure 4:**
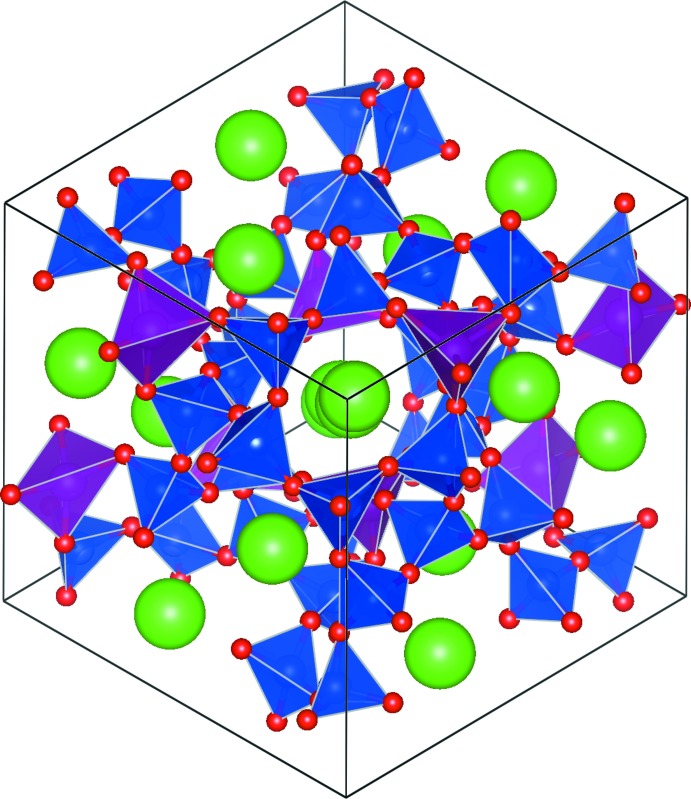
The crystal structure of Rb_2_MnSi_5_O_12_. Green spheres show Rb cations, blue polyhedra show SiO_4_ units, purple polyhedra show MnO_4_ units and red spheres represent O atoms.

Fig. 3 ▸ shows the Rietveld difference plot for Rb_2_MnSi_5_O_12_. The crystal structure of Rb_2_MnSi_5_O_12_ is displayed in Fig. 4 ▸ and consists of a framework of corner-sharing tetra­hedral SiO_4_ and MnO_4_ units; Rb cations sit in the extraframework channels. Note how inclusion of the larger Mn cation in the silicate framework compared to Ni causes the central channel of the crystal structure to be slightly more distorted for Rb_2_MnSi_5_O_12_ (Fig. 4 ▸) compared to Rb_2_NiSi_5_O_12_ (Fig. 2 ▸).

## Database survey   

Many different leucite analogue crystal structures are known at ambient temperature. Table 1 ▸ gives compositions, space groups, lattice parameters, and references for some known ambient temperature leucite crystal structures. In addition, a high-temperature structure for Cs_2_ZnSi_5_O_12_ in the space group *Pa* 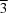 has been reported above 566 K (Bell & Henderson, 2012 ▸).

## Synthesis and crystallization   

The samples were made from stoichiometric mixtures of Rb_2_CO_3_, SiO_2_ and NiO (*X* = Ni) or MnO (*X* = Mn). These mixtures were ground together and then heated overnight at 873 K to decompose the carbonates, then melted in platinum crucibles at 1573 K for 1.5 h (*X* = Ni) or 1673 K for 2 h (*X* = Mn) before quenching to form glasses. The glasses were dry-crystallized at ambient pressure and 1193 K for 12 d.

## Refinement   

Crystal data, data collection and structure refinement details are summarized in Table 2 ▸. For each sample, a small amount of powder was ground and mounted on a low-background silicon wafer with a drop of acetone. These were mounted in flat-plate mode on a PANalytical X’Pert Pro MPD diffractometer. X-ray powder diffraction data were collected at 293 K using CuKα X-rays over the range 10–80°/2θ using a PANalytical X’Celerator area detector. The powder diffraction data collection time for each sample was 8 h 20 min.

All Bragg reflections in both of the powder diffraction patterns could be indexed in the space group *Pbca* with similar lattice parameters to that for the Cs_2_CdSi_5_O_12_ leucite (Bell *et al.*, 1994*a* ▸). The crystal structures (Bell & Henderson, 1996 ▸) of Cs_2_NiSi_5_O_12_ (*X* = Ni) and Cs_2_MnSi_5_O_12_ (*X* = Mn) were respectively used as starting models for Rietveld (1969 ▸) refinements. In both cases, Rb^+^ replaced Cs^+^ as the extraframework cation. Isotropic atomic displacement parameters were used for all atoms in these phases. In both refinements, the isotropic atomic displacement parameters were constrained to be the same for all sites occupied by the same element, each Rb site had the same displacement parameter as did each Si site and each O site. Soft constraints were used for both refinements, in both cases the Si—O distances were constrained to be 1.61±0.02 Å. For *X* = Ni, the Ni—O distances were constrained to be 1.88±0.02 Å, the mean Ni—O distance for Cs_2_NiSi_5_O_12_. For *X* = Mn, the Mn—O distances were constrained to be 1.98±0.02 Å, the mean Mn—O distance for Cs_2_MnSi_5_O_12_. In both cases, Si and *X* atoms were ordered onto separate tetra­hedrally coordinated sites, both refined structures are similar.

## Supplementary Material

Crystal structure: contains datablock(s) Rb2NiSi5O12, Rb2MnSi5O12, global. DOI: 10.1107/S2056989016001390/vn2106sup1.cif

Rietveld powder data: contains datablock(s) Rb2NiSi5O12. DOI: 10.1107/S2056989016001390/vn2106Rb2NiSi5O12sup5.rtv

CCDC references: 1449021, 1449020

Additional supporting information:  crystallographic information; 3D view; checkCIF report

## Figures and Tables

**Table 1:** Crystal structure parameters (Å°) for room-temperature leucite analogues

Stoichiometry	SG	*a*	*b*	*c*	β	*V*
K_2_MgSi_5_O_12_^*a*^	*Ia* 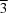 *d*	13.4190 (1)	13.4190 (1)	13.4190 (1)	90	2416.33 (5)
K_2_MgSi_5_O_12_^*a*^	*P*2_1_/*c*	13.168 (5)	13.652 (1)	13.072 (5)	91.69 (5)	2348 (2)
Cs_2_CdSi_5_O_12_^*b*^	*Pbca*	13.6714 (1)	13.8240 (1)	13.8939 (1)	90	2625.83 (6)
Cs_2_CuSi_5_O_12_^*c*^	*Pbca*	13.58943 (6)	13.57355 (5)	13.62296 (4)	90	2512.847 (13)
Cs_2_CuSi_5_O_12_^*c*^	*Ia* 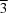 *d*	13.6322 (4)	13.6322 (4)	13.6322 (4)	90	2533.4 (2)
Cs_2_MgSi_5_O_12_^*d*^	*Pbca*	13.6371 (5)	13.6689 (5)	13.7280 (5)	90	2559.0 (2)
Rb_2_MgSi_5_O_12_^*d*^	*Pbca*	13.422 (1)	13.406 (1)	13.730 (1)	90	2470.6 (4)
Cs_2_ZnSi_5_O_12_^*d*^	*Pbca*	13.6415 (9)	13.6233 (8)	13.6653 (9)	90	2539.6 (3)
Rb_2_CdSi_5_O_12_^*e*^	*Pbca*	13.4121 (1)	13.6816 (1)	13.8558 (1)	90	2542.51 (5)
Cs_2_MnSi_5_O_12_^*e*^	*Pbca*	13.6878 (3)	13.7931 (3)	13.7575 (3)	90	2597.4 (2)
Cs_2_CoSi_5_O_12_^*e*^	*Pbca*	13.6487 (4)	13.7120 (4)	13.6828 (4)	90	2560.7 (2)
Cs_2_NiSi_5_O_12_^*e*^	*Pbca*	13.6147 (3)	13.6568 (5)	13.6583 (5)	90	2539.5 (1)
Rb_2_ZnSi_5_O_12_^*f*^	*Ia* 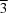 *d*	13.4972 (1)	13.4972 (1)	13.4972 (1)	90	2458.86 (3)
KFeSi_2_O_6_^*g*^	*I*4_1_/*a*	13.2207 (3)	13.2207 (3)	13.9464 (3)	90	2437.6 (2)
RbFeSi_2_O_6_^*g*^	*I*4_1_/*a*	13.4586 (1)	13.4586 (1)	13.9380 (1)	90	2524.63 (5)
CsFeSi_2_O_6_^*g*^	*Ia* 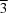 *d*	13.8542 (1)	13.8542 (1)	13.8542 (1)	90	2653.98 (3)
CsBSi_2_O_6_^*h*^	*I*4_1_/*a*	13.019 (2)	13.019 (2)	12.899 (3)	90	2186 (1)
CsAlSi_2_O_6_^*i*^	*Ia* 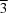 *d*	13.647 (3)	13.647 (3)	13.647 (3)	90	2541.6 (6)
KAlSi_2_O_6_^*j*^	*I*4_1_/*a*	13.09 (1)	13.09 (1)	13.75 (1)	90	2356 (5)
Cs_0.814_B_1.092_Si_1.977_O_6_^*k*^	*Ia-3d*	13.009 (8)	13.009 (8)	13.009 (8)	90	2202 (1)
Rb_2_MgSi_5_O_12_^*l*^	*Ia* 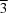 *d*	13.530 (1)	13.530 (1)	13.530 (1)	90	2476.8 (2)
Cs_2_BeSi_5_O_12_^*m*^	*Ia* 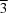 *d*	13.406 (1)	13.406 (1)	13.406 (1)	90	2409.3 (2)

Notes: SG = space group; all α and γ angles = 90°; (*a*) Bell *et al.* (1994*a* ▸); (*b*) Bell *et al.* (1994*b* ▸); (*c*) Bell *et al.* (2010 ▸); (*d*) Bell & Henderson (2009 ▸); (*e*) Bell & Henderson (1996 ▸); (*f*) Bell & Henderson (1994*a* ▸); (*g*) Bell & Henderson (1994*b* ▸); (*h*) Agakhanov *et al.* (2012 ▸); (*i*) Dimitrijevic *et al.* (1991 ▸); (*j*) Mazzi *et al.* (1976 ▸); (*k*) Bubnova *et al.* (2004 ▸); (*l*) Torres-Martinez & West (1986 ▸); (*m*) Torres-Martinez *et al.* (1984 ▸).

**Table 2:** Experimental details

	Rb_2_NiSi_5_O_12_	Rb_2_MnSi_5_O_12_
Crystal data		
Chemical formula	Rb_2_NiSi_5_O_12_	Rb_2_MnSi_5_O_12_
*M*_r_	562.06	557.92
Crystal system, space group	Orthorhombic, *P* *b* *c* *a*	Orthorhombic, *P* *b* *c* *a*
Temperature (K)	293	293
*a*, *b*, *c* (Å)	13.469 (3), 13.480 (3), 13.442 (2)	13.4085 (10), 13.6979 (11), 13.5761 (10)
*V* (Å^3^)	2440.7 (8)	2493.5 (3)
*Z*	8	8
Radiation type	Cu *K*α, λ = 1.540560 Å	Cu *K*α, λ = 1.540560 Å
Specimen shape, size (mm)	Irregular, 10 × 10	Irregular, 10 × 10

Data collection		
Diffractometer	PANalytical X’Pert Pro MPD	PANalytical X’Pert Pro MPD
Specimen mounting	Flat plate	Flat plate
Data collection mode	Reflection	Reflection
Scan method	Step	Step
2θ values (°)	2θ_min_ = 9.897 2θ_max_ = 79.883 2θ_step_ = 0.017	2θ_min_ = 10.139 2θ_max_ = 80.125 2θ_step_ = 0.017

Refinement		
*R* factors and goodness of fit	*R*_p_ = 9.048, *R*_wp_ = 12.007, *R*_exp_ = 4.263, *R*_Bragg_ = 10.421, χ^2^ = 7.935	*R*_p_ = 6.527, *R*_wp_ = 8.847, *R*_exp_ = 3.909, *R*_Bragg_ = 5.989, χ^2^ = 5.121
No. of parameters	73	71
No. of restraints	24	24

Computer programs: *X’Pert Data Collector* (PANalytical, 2006 ▸), *FULLPROF* (Rodríguez-Carvajal, 1993 ▸), *VESTA* (Momma & Izumi, 2008 ▸) and *publCIF* (Westrip, 2010 ▸).

## supplementary crystallographic information

### Crystal data

**Table:**

Rb_2_MnSi_5_O_12_	*Z* = 8
*M**_r_* = 557.92	*D*_x_ = 2.975 (1) Mg m^−^^3^
Orthorhombic, *P**b**c**a*	Cu *K*α radiation, λ = 1.540560 Å
Hall symbol: -P 2ac 2ab	*T* = 293 K
*a* = 13.4085 (10) Å	pale brown-pink
*b* = 13.6979 (11) Å	irregular, 10 × 10 mm
*c* = 13.5761 (10) Å	Specimen preparation: Prepared at 1193 K and 100 kPa
*V* = 2493.5 (3) Å^3^	

### Data collection

**Table:**

PANalytical X'Pert Pro MPD diffractometer	Data collection mode: reflection
Radiation source: X-ray tube	Scan method: step
Specimen mounting: flat plate	2θ_min_ = 10.139°, 2θ_max_ = 80.125°, 2θ_step_ = 0.017°

### Refinement

**Table:**

*R*_p_ = 6.527	4189 data points
*R*_wp_ = 8.847	Profile function: T-C-H Pseudo-Voigt
*R*_exp_ = 3.909	71 parameters
*R*_Bragg_ = 5.989	24 restraints
χ^2^ = 5.121	Background function: cubic splines interpolation

### Fractional atomic coordinates and isotropic or equivalent isotropic displacement parameters (Å^2^)

**Table:**

	*x*	*y*	*z*	*U*_iso_*/*U*_eq_	
Rb1	0.1311 (8)	0.1348 (8)	0.1535 (6)	0.0544 (19)*	
Rb2	0.3811 (10)	0.3968 (7)	0.3953 (7)	0.0544 (19)*	
Mn1	0.3707 (9)	0.8342 (7)	0.9432 (6)	0.00127*	
Si2	0.1328 (11)	0.6601 (10)	0.6011 (10)	0.024 (2)*	
Si3	0.5921 (11)	0.1146 (10)	0.6255 (10)	0.024 (2)*	
Si4	0.6449 (10)	0.5993 (11)	0.1054 (10)	0.024 (2)*	
Si5	0.9006 (10)	0.3583 (11)	0.8208 (10)	0.024 (2)*	
Si6	0.8312 (11)	0.9317 (10)	0.3329 (11)	0.024 (2)*	
O1	0.4758 (12)	0.355 (3)	0.1607 (19)	0.00127*	
O2	0.083 (2)	0.4973 (12)	0.399 (3)	0.00127*	
O3	0.397 (2)	0.150 (3)	0.4890 (13)	0.00127*	
O4	0.7328 (12)	0.4230 (19)	0.620 (2)	0.00127*	
O5	0.659 (2)	0.7326 (14)	0.360 (2)	0.00127*	
O6	0.363 (4)	0.632 (2)	0.7605 (17)	0.00127*	
O7	0.9822 (15)	0.877 (3)	0.645 (2)	0.00127*	
O8	0.659 (3)	0.9530 (13)	0.860 (3)	0.00127*	
O9	0.893 (2)	0.635 (2)	0.9119 (13)	0.00127*	
O10	0.2099 (14)	0.9281 (18)	0.135 (3)	0.00127*	
O11	0.132 (3)	0.1885 (12)	0.9545 (17)	0.00127*	
O12	0.892 (3)	0.139 (3)	0.2068 (12)	0.00127*	

### Geometric parameters (Å, º)

**Table:**

Rb1—O1^i^	4.09 (4)	Rb2—O10^i^	3.76 (4)
Rb1—O2^ii^	3.51 (3)	Rb2—O11^iii^	3.63 (4)
Rb1—O2^iii^	3.95 (4)	Rb2—O12^iv^	3.80 (4)
Rb1—O3^iv^	3.69 (3)	Mn1—O4^xvii^	2.04 (2)
Rb1—O4^v^	3.46 (3)	Mn1—O7^xvi^	2.00 (3)
Rb1—O5^vi^	3.12 (3)	Mn1—O9^xviii^	2.03 (2)
Rb1—O6^vii^	3.51 (3)	Mn1—O11^xix^	2.002 (19)
Rb1—O7^viii^	3.13 (3)	Si2—O1^xx^	1.68 (2)
Rb1—O8^viii^	3.07 (4)	Si2—O3^xix^	1.58 (2)
Rb1—O9^viii^	3.29 (3)	Si2—O5^xii^	1.60 (2)
Rb1—O10^ix^	3.03 (3)	Si2—O10^xxi^	1.66 (3)
Rb1—O11^x^	2.80 (2)	Si3—O1^xxii^	1.68 (2)
Rb1—O12^xi^	3.29 (4)	Si3—O2^xxiii^	1.57 (2)
Rb1—O12^iv^	3.98 (4)	Si3—O6^xiv^	1.68 (3)
Rb2—O1	3.48 (3)	Si3—O11^xxiv^	1.58 (3)
Rb2—O2	4.23 (3)	Si4—O2^xxv^	1.63 (2)
Rb2—O3	3.62 (4)	Si4—O3^xxvi^	1.56 (3)
Rb2—O4^viii^	2.91 (3)	Si4—O4^xv^	1.68 (2)
Rb2—O5^vi^	4.17 (3)	Si4—O12^xxvii^	1.56 (3)
Rb2—O5^viii^	3.80 (3)	Si5—O5^xxviii^	1.57 (3)
Rb2—O6^vii^	3.77 (5)	Si5—O7^xxix^	1.66 (3)
Rb2—O6^viii^	4.05 (5)	Si5—O8^xiii^	1.61 (3)
Rb2—O7^xii^	3.43 (4)	Si5—O12^xxii^	1.55 (2)
Rb2—O7^xiii^	3.86 (3)	Si6—O6^xxx^	1.60 (3)
Rb2—O8^xiv^	3.45 (4)	Si6—O8^xxxi^	1.63 (2)
Rb2—O9^xv^	3.07 (3)	Si6—O9^xxxii^	1.63 (3)
Rb2—O9^xvi^	4.19 (3)	Si6—O10^xxv^	1.68 (3)
			
O4^xvii^—Mn1—O7^xvi^	94.7 (15)	O5^xxviii^—Si5—O7^xxix^	121 (3)
O4^xvii^—Mn1—O9^xviii^	112.6 (18)	O5^xxviii^—Si5—O8^xiii^	106 (2)
O4^xvii^—Mn1—O11^xix^	128.0 (19)	O5^xxviii^—Si5—O12^xxii^	109 (3)
O7^xvi^—Mn1—O9^xviii^	114.1 (18)	O7^xxix^—Si5—O8^xiii^	105 (3)
O7^xvi^—Mn1—O11^xix^	111 (2)	O7^xxix^—Si5—O12^xxii^	110 (3)
O9^xviii^—Mn1—O11^xix^	97.8 (16)	O8^xiii^—Si5—O12^xxii^	106 (3)
O1^xx^—Si2—O3^xix^	104 (2)	O6^xxx^—Si6—O8^xxxi^	134 (3)
O1^xx^—Si2—O5^xii^	98 (2)	O6^xxx^—Si6—O9^xxxii^	94 (2)
O1^xx^—Si2—O10^xxi^	109 (2)	O6^xxx^—Si6—O10^xxv^	117 (3)
O3^xix^—Si2—O5^xii^	117 (3)	O8^xxxi^—Si6—O9^xxxii^	111 (2)
O3^xix^—Si2—O10^xxi^	111 (3)	O8^xxxi^—Si6—O10^xxv^	93 (2)
O5^xii^—Si2—O10^xxi^	116 (2)	O9^xxxii^—Si6—O10^xxv^	108 (2)
O1^xxii^—Si3—O2^xxiii^	103 (3)	Si2^vii^—O1—Si3^iii^	134.2 (18)
O1^xxii^—Si3—O6^xiv^	92 (3)	Si3^v^—O2—Si4^iv^	142.2 (18)
O1^xxii^—Si3—O11^xxiv^	111 (3)	Si2^i^—O3—Si4^vi^	137.6 (19)
O2^xxiii^—Si3—O6^xiv^	111 (3)	Mn1^xiv^—O4—Si4^xxviii^	120.5 (14)
O2^xxiii^—Si3—O11^xxiv^	120 (2)	Si2^xxx^—O5—Si5^xv^	136.4 (19)
O6^xiv^—Si3—O11^xxiv^	115 (2)	Si3^xvii^—O6—Si6^xii^	138 (2)
O2^xxv^—Si4—O3^xxvi^	100 (3)	Mn1^xxiv^—O7—Si5^xxxiii^	146.0 (16)
O2^xxv^—Si4—O4^xv^	110 (2)	Si5^xxvii^—O8—Si6^xxxiv^	138.2 (19)
O2^xxv^—Si4—O12^xxvii^	100 (3)	Mn1^xxxv^—O9—Si6^xxi^	132.5 (15)
O3^xxvi^—Si4—O4^xv^	122 (2)	Si2^xxxii^—O10—Si6^iv^	134.1 (18)
O3^xxvi^—Si4—O12^xxvii^	117 (2)	Mn1^i^—O11—Si3^xvi^	125.5 (15)
O4^xv^—Si4—O12^xxvii^	106 (3)	Si4^xiii^—O12—Si5^iii^	155.7 (19)

Symmetry codes: (i) −*x*+1/2, *y*−1/2, *z*; (ii) −*x*, *y*−1/2, −*z*+1/2; (iii) *x*, −*y*+1/2, *z*−1/2; (iv) *x*−1/2, *y*, −*z*+1/2; (v) *x*−1/2, −*y*+1/2, −*z*+1; (vi) −*x*+1, *y*−1/2, −*z*+1/2; (vii) −*x*+1/2, −*y*+1, *z*−1/2; (viii) −*x*+1, −*y*+1, −*z*+1; (ix) *x*, *y*−1, *z*; (x) *x*, *y*, *z*−1; (xi) *x*−1, *y*, *z*; (xii) *x*−1/2, −*y*+3/2, −*z*+1; (xiii) −*x*+3/2, *y*−1/2, *z*; (xiv) −*x*+1, *y*−1/2, −*z*+3/2; (xv) −*x*+3/2, −*y*+1, *z*−1/2; (xvi) *x*−1/2, *y*, −*z*+3/2; (xvii) −*x*+1, *y*+1/2, −*z*+3/2; (xviii) *x*−1/2, −*y*+3/2, −*z*+2; (xix) −*x*+1/2, *y*+1/2, *z*; (xx) −*x*+1/2, −*y*+1, *z*+1/2; (xxi) *x*, −*y*+3/2, *z*+1/2; (xxii) *x*, −*y*+1/2, *z*+1/2; (xxiii) *x*+1/2, −*y*+1/2, −*z*+1; (xxiv) *x*+1/2, *y*, −*z*+3/2; (xxv) *x*+1/2, *y*, −*z*+1/2; (xxvi) −*x*+1, *y*+1/2, −*z*+1/2; (xxvii) −*x*+3/2, *y*+1/2, *z*; (xxviii) −*x*+3/2, −*y*+1, *z*+1/2; (xxix) −*x*+2, *y*−1/2, −*z*+3/2; (xxx) *x*+1/2, −*y*+3/2, −*z*+1; (xxxi) −*x*+3/2, −*y*+2, *z*−1/2; (xxxii) *x*, −*y*+3/2, *z*−1/2; (xxxiii) −*x*+2, *y*+1/2, −*z*+3/2; (xxxiv) −*x*+3/2, −*y*+2, *z*+1/2; (xxxv) *x*+1/2, −*y*+3/2, −*z*+2.
